# Glances at the Hospitals

**Published:** 1904-07-30

**Authors:** 


### GLANCES AT THE HOSPITALS.

### ROYAL UNITED HOSPITAL, BATH.

THE income amounted to £5,774, and the expenditure to £10,071. There was a balance due to the treasurers of £4,297. The subscriptions amounted to £1,727, and the workmen's collections to £1,111. The state of the finances has caused some anxiety to the managers; a sub-committee has been constantly sitting, and through their advice a considerable saving in expenditure has resulted, but an additional £1,000 a year is required before the ordinary income equals the ordinary expenditure. At the beginning of the year there were 76 patients in residence, and 1,313 were admitted during it, making a total of 1,389. Of these 1,117 were discharged recovered or relieved, 72 not relieved, 110 died, and 90 remained under treatment. The medical and surgical tables are very clearly drawn out. Of 36 cases of pneumonia 26 recovered, 6 died, and 4 remained in the hospital on the date of the report. There were 20 cases of pleurisy and all recovered. The operation table does not give results; it merely states the number operated on for various causes, and it might as well have been omitted. The anæsthetic table shows that gas was administered 346 times, chloroform 277 times, gas and ether 179 times, gas, ether and chloroform 48 times, ether 47 times, A.C.E. mixture 15 times, and other agents 10 times. We failed to notice any statement of the cost per head, or of the cost per bed occupied, or of the average stay in the hospital.

### VICTORIA INFIRMARY, TYNEMOUTH.

THE income for the year was £1,925, and the expenditure was £1,892. This balance increased by one left over from the previous year enabled the committee to close the year's work with a sum in hand of £109. This shows careful management; and it is such institutions that deserve to be supported by the public. The in-patients numbered 448, and of these 349 were cured, 49 were relieved, 25 died, and 25 remained under treatment. The death-rate was only 5 5 per cent., and if patients dying within 48 hours of admission be left out of the calculation, it would fall to 2 9 per cent. There is no anæsthetic table given; but the medical and surgical tables contain all necessary information as to the results of treatment.

